# Fusel Oil

**Published:** 1853-01

**Authors:** 


*Fusel Oil.* — Our readers will recollect a communication from Dr. C. T. Jackson, in which the occasional fatal effects of chloroform were attributed to Fusel oil becoming commingled with it in its preparation. By the N. Y. Med. Times we perceive that at the monthly meeting of the American Academy of Arts and Sciences, held at Boston, September 14, experiments with Dr. Jackson's Fusel oil compounds on animals were reported, the conclusions from which are: 1. That the vapor of the Fusel oil compound alone is not poisonous; 2. That the view that this compound is the cause of the occasional fatal effects of chloroform is not sustained.

